# Keeping the Feast at Salisbury Infirmary

**Published:** 1913-01-04

**Authors:** 


### KEEPING THE FEAST AT SALISBURY INFIRMARY.

However much the Insurance Act may ultimately affect the voluntary hospitals, there was at any rate no diminution in the measure of help given to provide

Christmas festivities for the patients at Salisbury Infirmary, even though a larger number of patients remained under treatment during the season than has been the case for many years past. The customary "Appeal" which is sent out in the joint names of the Matron and Chaplain early in December, asking for help to provide a tree and other festivities, was very generously responded to, the donors representing every section of the community, and including many past patients and former members of the staff. Gifts in money and kind arrived from all parts of the country and city, and provision on a liberal scale was able to be made, without entailing extra expense on the general fund.

Add to this the whole-hearted service that was rendered by one and all, from senior members of the staff to the youngest wardmaid, and one can understand why the true spirit of Christmas was felt and recognised by all. It has always been the custom to bring the Church festival into prominence on the day itself, reserving the entertainments for succeeding days, and this rule was again followed. Christmas hymns were sung by the nursing staff in the early morning. Two celebrations of the Holy Communion were held. The chapel had been tastefully decorated, the evergreens and flowers used having been amongst the gifts for Christmas. The decorations in the wards were the expression of much taste and skill on the part of the nursing staff and the house surgeons. The scheme of colour was different in each ward, although holly of exceptional beauty was abundant everywhere. The lamp-shades were quite works of art, and were much admired. Every young child had a stocking full of toys on Christmas morning. The other patients each had some small gift, but the large presents were reserved for the "tree" parcels.

Early in the afternoon a large number of invited guests arrived at the hospital, each patient having had the privilege of inviting two friends. Tea-tables, bountifully laden with good things and tastefully decorated, were the centre of attraction in each ward, the teas to the patients and their friends being the gift of the respective ward visitors who presided personally at the tea-table, giving up the whole afternoon for the purpose. While tea was in progress many other visitors appeared—the Bishop, Dr. Ridgeway, the City M.P., the Mayor and Mayoress, the visiting staff and their friends—and went from ward to ward with greetings for everyone. After tea carols were sung in each ward by a choir composed of nurses and maids under the direction of the Chaplain. This gave great pleasure to all the patients. Two entertainments were given on succeeding days, one by a member of the visiting staff and his friends, and one by friends of one of the ward visitors. The distribution of gifts from the Christmas tree took place on Friday, and gave immense pleasure. The magnificent tree (also a gift) was laden with substantial parcels, each patient receiving useful articles of clothing, with toys for the children, books, games, and fancy articles for older patients.

Every member of the staff also received a really nice gift. The parcels were wrapped in variously tinted paper, with toys outside, and looked very attractive, and trimmings of every description made the whole resplendent. The tree was 15 feet high, every branch strengthened with iron rods to support the weight of parcels. It was illuminated by electricity. Small child-patients dressed in white and scarlet were placed in their cots at the nearest approach to the tree, and claimed much attention from the many visitors who were present. The evening concluded with music and refreshments for the patients, and a never-to-be-forgotten day came to an end.

